# Comparison of 99mTc radiolabeled somatostatin antagonist with [^68^ Ga]Ga-DOTA-TATE in a patient with advanced neuroendocrine tumor

**DOI:** 10.1007/s00259-023-06335-9

**Published:** 2023-07-15

**Authors:** Marta Opalinska, Luka Lezaic, Clemens Decristoforo, Petra Kolenc, Renata Mikolajczak, Andrej Studen, Urban Simoncic, Irene Virgolini, Malgorzata Trofimiuk-Muldner, Piotr Garnuszek, Christine Rangger, Melpomeni Fani, Boguslaw Glowa, Konrad Skorkiewicz, Alicja Hubalewska-Dydejczyk

**Affiliations:** 1 Chair and Department of Endocrinology, Jagiellonian University Medical College, Krakow, Poland; 2 Department of Nuclear Medicine, University Medical Centre Ljubljana, Ljubljana, Slovenia; 3 Faculty of Medicine, University of Ljubljana, Ljubljana, Slovenia; 4 Department of Nuclear Medicine, Medical University Innsbruck, Innsbruck, Austria; 5 Faculty of Pharmacy, University of Ljubljana, Ljubljana, Slovenia; 6 Radioisotope Centre POLATOM, National Centre for Nuclear Research, Otwock, Poland; 7 Faculty of Mathematics and Physics, University of Ljubljana, Ljubljana, Slovenia; 8 Jožef Stefan Institute, Ljubljana, Slovenia; 9 Universitätsspital Basel, Basel, Switzerland; 10 University Hospital Krakow, Krakow, Poland

Gallium-68-labelled somatostatin analogues are a gold standard of neuroendocrine tumors (NETs) PET imaging being a tool for personalized treatment. However, in some cases of low somatostatin receptor (SSTR) expressing tumors, its clinical value can be limited. SSTR antagonists in comparison to currently used agonistic analogues recognize more binding sites on NET cells [1], which may improve the diagnostic efficacy enabling more precise staging leading to the better outcome.

SPECT radiopharmaceuticals represent the cornerstone of molecular imaging due to their wide availability [2] and the development of a radiopharmaceutical based on technetium-99 m-labeled SSTR antagonist would improve access to such clinically feasible diagnostic tool.

Below (Fig. 1), we present a comparison of SPECT/CT with a SSTR antagonist [^99m^Tc]Tc-TECANT1 (N4-p-Cl-Phe-cyclo(D-Cys-Tyr-D-Aph(Cbm)-Lys-Thr-Cys)-D-Tyr-NH2, where D-Aph(Cbm): D-4-amino-carbamoyl-phenylalanine) [3] and [^68^ Ga]Ga-DOTA-TATE PET/CT (EudraCT no 2019–003379-20). Please note better visualization of the lesions seen in the study with [^99m^Tc]Tc-TECANT1 (in 6 out of 7 lesions) as well as significantly higher tumor-to-background ratio (in primary and metastatic lesions) obtained with the novel tracer (measured as an absolute number of counts in lesion to background (TBR)) in comparison to [^68^ Ga]Ga-DOTA-TATE PET/CT (4,07 (range 1,36–7,58) vs 2,26 (range 1,15–3,39)).


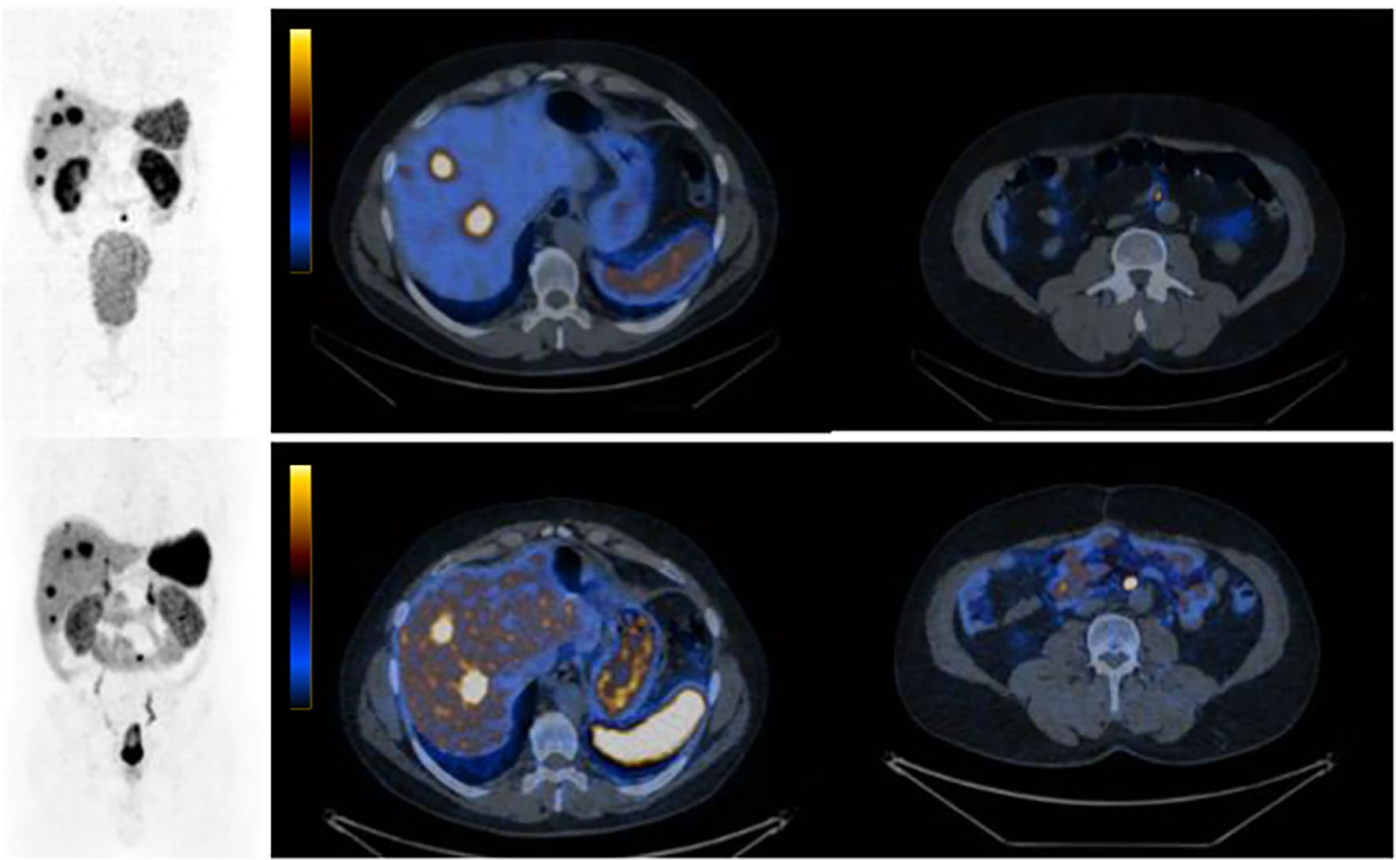


Fig. 1 Maximum intensity projection (MIP) and axial fused images: upper line -[^99m^Tc]Tc-TECANT1 SPECT/CT (4 h post injection, injected activity 785 MBq, 120 frames, 20 s per frame); bottom line — [^68^ Ga]Ga-DOTA-TATE PET/CT (1 h post injection, injected activity 146 MBq; 3 min per bed). Scans were obtained within 13 days. Long-acting somatostatin analogue was withdrawn 4 weeks before imaging

Although PET is considered more sensitive than SPECT [4], the presented new radiopharmaceutical holds promise to provide higher TBR values compared to the current gold standard ^68^ Ga-DOTA-TATE PET/CT. In combination with the development of quantitative SPECT imaging of NETs, the use of ^99m^Tc-labelled SSTR antagonists could provide a widely available, clinically significant step in the personalized management of NETs.

## Data Availability

The original contributions presented in the study are included in the article. Further inquiries can be directed to the corresponding author.
